# Using machine learning techniques to develop risk prediction models to predict graft failure following kidney transplantation: protocol for a retrospective cohort study

**DOI:** 10.12688/f1000research.20661.2

**Published:** 2020-03-09

**Authors:** Sameera Senanayake, Adrian Barnett, Nicholas Graves, Helen Healy, Keshwar Baboolal, Sanjeewa Kularatna

**Affiliations:** 1 Australian Center for Health Service Innovation, Queensland University of Technology, Kelvin Grove, QLD, 4059, Australia; 2 Royal Brisbane Hospital for Women, Brisbane, QLD, 4001, Australia; 3 School of Medicine, University of Queensland, Brisbane, QLD, 4001, Australia

**Keywords:** Machine learning, Risk prediction models, Kidney transplant, Graft failure

## Abstract

**Background: **A mechanism to predict graft failure before the actual kidney transplantation occurs is crucial to clinical management of chronic kidney disease patients.  Several kidney graft outcome prediction models, developed using machine learning methods, are available in the literature.  However, most of those models used small datasets and none of the machine learning-based prediction models available in the medical literature modelled time-to-event (survival) information, but instead used the binary outcome of failure or not. The objective of this study is to develop two separate machine learning-based predictive models to predict graft failure following live and deceased donor kidney transplant, using time-to-event data in a large national dataset from Australia.

**Methods: **The dataset provided by the Australia and New Zealand Dialysis and Transplant Registry will be used for the analysis. This retrospective dataset contains the cohort of patients who underwent a kidney transplant in Australia from January 1 ^st^, 2007, to December 31 ^st^, 2017. This included 3,758 live donor transplants and 7,365 deceased donor transplants. Three machine learning methods (survival tree, random survival forest and survival support vector machine) and one traditional regression method, Cox proportional regression, will be used to develop the two predictive models (for live donor and deceased donor transplants). The best predictive model will be selected based on the model’s performance.

**Discussion: **This protocol describes the development of two separate machine learning-based predictive models to predict graft failure following live and deceased donor kidney transplant, using a large national dataset from Australia. Furthermore, these two models will be the most comprehensive kidney graft failure predictive models that have used survival data to model using machine learning techniques. Thus, these models are expected to provide valuable insight into the complex interactions between graft failure and donor and recipient characteristics.

## Introduction

The prevalence of chronic kidney disease is increasing globally. Along with this increment, the number of patients in end-stage of renal disease (ESRD) and the demand for kidney transplantation, along with other renal replacement therapies, have increased over recent years ^1, 2^. Compared with available renal replacement therapies, renal transplantation has dramatically improved the quality of life and the survival rate of patients with ESRD. However, evidence indicate that the health systems around the world, have failed to meet the increasing demand for kidney grafts. This is evident from the growing prevalence of ESRD in the world ^3^. Further, kidney transplants pose a significant cost burden to health systems compared with other treatment modalities, as they consume a large amount of resources.

It is important that the donor kidneys are transplanted to the most suitable recipients in order to minimise the number of graft failures, and thus minimise the number of patients returning to the already-burdened waiting list ^4^. However, evidence indicates that the incidence of graft failure following kidney transplantation has increased over the years, possibly owing to increased transplantation of kidneys from expanded-criteria donors and donors after cardiac death, who are more prone to graft failure ^5^. Graft failure is associated with prolonged hospital stay and higher health system costs ^6, 7^. A mechanism to predict graft failure before the actual transplantation occurs is crucial in this regard. Similar predictive models have been increasingly used in the recent past, and these have assisted clinicians with evidence-based medical decision-making ^4, 8– 10^. Numerous conventional predictive models are available in the literature to predict the graft loss among kidney transplant patients ^11– 14^. Interestingly, novel techniques based on machine learning methods provide the potential to produce more favourable results ^15^.

Machine learning techniques have been used to develop kidney graft outcome-prediction models ^4, 8– 10, 16^. With the exception of the prediction models developed in the United States ^4, 10, 17– 19^, most of the other prediction models have been developed using datasets with less than 1,000 records. However, evidence indicates that large sample sizes lead to better prediction accuracy in machine learning-based prediction models ^20^. The model developed by Akl *et al*. (2008) ^8^, using 1,900 live donor transplant records from a single urology centre in Egypt, is the only machine learning predictive model available that is based exclusively on live donor transplants, while most of the other models are based either exclusively on deceased donor transplants, or both deceased and living donor transplants. However, evidence indicates that the graft failure rate and the predictors of graft failure significantly differ between live and deceased donor transplants ^5^. Therefore, from a clinical perspective, two separate valid and reliable prediction models, i.e. live and deceased donor transplants, would give superior clinical utility.

Time-to-event (survival) information had not been modelled in any of the machine learning based prediction models available in the medical literature. Instead, most have used the binary outcome of failure or not as the outcome variable. However, presence of censored observations makes predictions done using this type of prediction models less accurate. Therefore, incorporating the timing of the event to the prediction model, could lead to better prediction models ^21^.

In this background. the objective of this study is to develop two separate machine learning-based predictive models to predict graft failure following live and deceased donor kidney transplant, using time-to-event (survival) data in a large national dataset from Australia.

## Protocol

This study will evaluate different machine learning methods in predicting kidney graft failure.

### Study cohort

The dataset was provided by the Australia and New Zealand Dialysis and Transplant Registry (ANZDATA). ANZDATA collects and reports the incidence, prevalence and outcome of dialysis treatment and kidney transplantation for patients with end-stage kidney disease across Australia and New Zealand. The retrospective dataset contains the cohort of patients who underwent a kidney transplant in Australia from January 1 ^st^, 2007, to December 31 ^st^, 2017. This included 3,758 live donor transplants and 7,365 deceased donor transplants.

Two separate predictive models will be developed for live donor and deceased donor transplants using separate datasets for live and deceased donor transplants.

### Outcome

Graft survival of the most recent kidney transplants will be converted to a binary variable and will be the primary outcome. Patients who died with a functioning graft will not be considered positive for graft failure, but will be included in all models and censored at their death date. The time to the graft failure will be calculated in days from the date of transplantation. If the outcome of interest has not happened within the time period the data is available (2007 to 2017), it will be considered as right-censored with a time from the date of transplantation to the censoring date. In total, n=65 (0.9%) patients in the deceased donor dataset (n=7,365) and n=73 (1.9%) patients in the living donor dataset (n=3,758) have been lost to follow-up. Their records will be right-censored from the last date where the follow-up information is available.

### Independent variables

The data consist of de-identified recipient and donor characteristics of the transplants. In all, 83 possible variables were identified as potential risk factors for graft failure (Supplementary material) ^22^.

### Model development

Three machine learning methods and one traditional regression method, Cox proportional regression, will be used to develop the two separate predictive models, i.e. one for live donor and one for deceased donor transplants. The machine learning methods that will be used are: survival tree ^23^, random survival forest ^24^ and survival support vector machine ^25^. Thus, each prediction model will be developed using four methods, and the best predictive model will be selected based on the model’s performance, as described in a later section.

**Figure 1.**
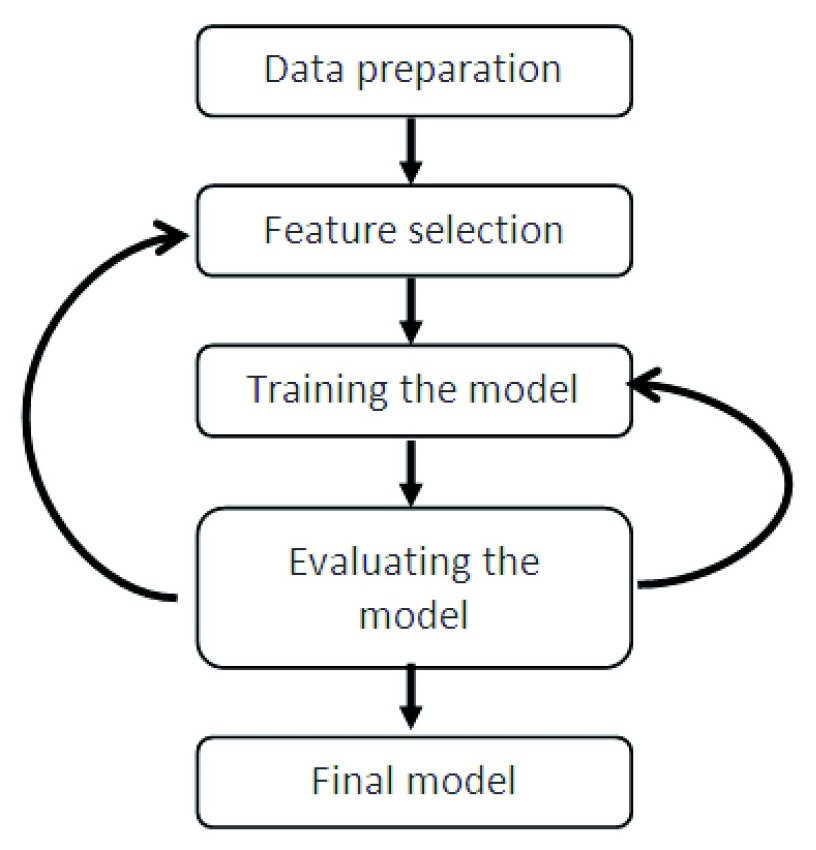
Model development process.

Model development is a systematic process which involves five steps, as indicated in Figure 1.

***Data preparation.*** Data preparation involves several steps, such as data cleaning, handling text and categorical attributes, and feature scaling.

Retrospective datasets have the inherent property of having missing values, and most machine learning algorithms do not work well with missing values. Depending on the extent the missing values in each of the variables, the decision will be made to either exclude the particular variable, categorise the missing values as a separate category, or use an imputation method to impute the missing values.

Machine learning algorithms work well with numerical arrays compared with text (e.g. the donor’s cause of death: traumatic brain injury, cerebral infarct and intracranial haemorrhage). Thus, text variables will be categorised appropriately; each category will be assigned a numerical variable (e.g., traumatic brain injury will be assigned a ‘1’, cerebral infarct a ‘2’, and intracranial haemorrhage a ‘3’). However, when text variables are converted to numeric categories, the machine learning algorithms assume that two nearby values are more similar than two distant values (e.g. ‘1’ is more similar to ‘2’ than ‘3’). To overcome this, the categorical variables will be dummy coded into nominal categories.

With a few exceptions, machine learning algorithms do not perform well when numerical input variables are not in the same scale (e.g., donor age ranges from 9 to 87, while the donor serum creatinine value ranges from 3 to 857). The numerical input variables will be standardised to convert them all to the same scale before applying the different machine learning methods ^26^. Parameter estimates and plots will be transformed back to the original scale as this will be the most useful scale for clinicians.

Collinearity in the independent variables will be assessed using variance inflation factor (VIF) ^27^. VIF of more than four will be considered as presence of collinearity and one of the correlated variables will be excluded from the analysis and the models re-run and re-validated.

***Feature selection.*** Feature (variable) selection is the process of selecting the most relevant variables that should be included in the model. We will carefully select a potentially large set variables to be used by the feature selection methods in discussion with clinical colleagues. We will use variable selection methods that aim to create a parsimonious set of predictor variables from the larger set using cross-validation. We will reflect on the variables selected with our clinical colleagues to verify that the model makes clinical sense. Since the predictive models might potentially be used in pre-transplant decision making, only variables available before transplantation will be used in developing prediction models.

Three methods will be used for feature selection:

1. *Medical literature and expert opinion.* Studies done on kidney transplant graft survival will be reviewed to identify the significant predictors of graft survival. The identified list will be validated by clinical experts. The variables identified as potentially important in this step will be included in the predictive models.
2. *Principal component analysis* ^28^. This method of feature selection will be performed using exploratory factor analysis using principal component analysis. Bartlett’s test of sphericity and the Kaiser-Meyer-Olkin measure will be performed to assess the factorability of the data. Factor structure and factor loadings after varimax rotation will be assessed. The selection of factors will be done, depending on the eigenvalues. The factors are considered relevant if the eigenvalues are more than one. The input variables will be observed for their factor coefficients, and more than 0.4 will be considered as well loaded and will be used for the model development. However, variables that have factor coefficients of less than 0.4 for all the factors will be excluded from the model development.
*Elastic net.* Elastic net uses both Lasso and Ridge regression to select features ^29^. These two regularisation methods minimise the sum of squared residuals using L1 and L2 norms to limit the size of coefficients in the model ^30^. The ideal size of the penalty will be selected using cross-validation. A stronger penalty is a tighter “lasso” that means fewer independent variables are selected. We will examine the plot of variable estimates against the penalty term to understand how the independent variables interact.

Possible combinations of the three sets of selected features from the three different feature selection mechanisms (i.e., medical literature and expert opinion, principal component analysis and elastic net) will be considered as input variables for the four methods of predictive models and the four methods of machine learning algorithms. Seven possible combinations are indicated in Table 1. The best set of input variables for each of the predictive models will be selected based on the model’s performance.

**Table 1.** Possible combinations of input variable groups.

Combination No.	Selected input variable group
Combination 1	ML & EO
Combination 2	PCA
Combination 3	EN
Combination 4	ML & EO and PCA
Combination 5	ML & EO and EN
Combination 6	PCA and EN
Combination 7	ML & EO, PCA and EN

ML & EO: Medical literature and expert opinion; PCA : Principal component analysis; EN : Elastic net

***Model training.*** During model training the dataset is randomly divided in to two parts: a training dataset and validation dataset. This prevents over-fitting and provides models that are more robust and give more realistic predictions of their prediction accuracy. Several spilt proportions have been used in models in relevant literature, such as 90:10% and 80:20%, with 70:30% being the most common ^31^. Thus, in the present study dataset will be split in to two parts, with 70% of the data to train the model and 30% to validate the developed models. Given our large sample size we expect that this approach would produce similar results to multiple cross-validations. However, in live donor transplant sample of around 3,758 we will use use cross-validation to estimate the variability in our model evaluation statistics, and if the variability is large (more than 10% of the mean accuracy) then we will use cross-validation for this sample.

Since the outcome of interest is a survival function, the training dataset will be fitted to following models; Cox proportional regression method, survival tree, random survival forest and survival support vector machine. The R programming package, specifically the packages Survivalsvm, Ranger, Survival and LTRCtrees, will be used to develop all the predictive models ^32^.

1. Cox proportional regression ^33^
  Cox proportional regression method is a semi-parametric model which is often used to explore the relationship between time-to-event data and several explanatory variables. This method assumes that effects of the different variables on survival are constant over time and are additive in a particular scale.
2. Survival Tree ^23^
  A survival tree is a tree-like structure, where leaves represent outcome variables, i.e. graft failure (1) or no graft failure (0), and branches represent conjunctions of input variables that produced the outcome. Based on the chosen split criterion (survival statistic), a survival tree divides the data (parent node) into two groups (child nodes). The two resulting groups become the new parent nodes and are subsequently divided further into two child nodes based on the characteristics of the input variables.
  Hyper-parameters will be regularised until the optimal decision tree is created. The hyper-parameters include maximum depth of the decision tree, minimum number of samples a node must have before it can be split, and the minimum number of samples a node must have.
  Trees are often useful for identifying important interactions between independent variables. If strong interactions are found by the tree, then these may be added as additional independent variables to the other approaches as this could increase the models’ predictive ability.
3. Random survival forest ^24^
  Random forest is an ensemble method in machine learning where multiple unpruned survival trees are constructed via bootstrap aggregation ^34, 35^. Each tree predicts a classification independently and the final prediction is made based on the class (i.e. graft failure versus no graft failure) that gets the most “votes” ^36, 37^. This method of aggregation of multiple survival trees has several advantages: the prediction is resistant to outliers, less noisy and suitable for small datasets ^38^.
  The following hyper-parameters will be regularised until the optimal prediction is made: number of survival tree classifiers and maximum number of nodes.
4. Survival support vector machine ^25^
  Survival support vector machine is a well-suited method to classify complex but small or medium-sized datasets. Survival support vector machine uses hyperplanes to classify different classes and achieves high predictive accuracy when the data is linearly separable (linear kernel function). However, kernel functions (i.e. Gaussian, sigmoid and polynomial) can be used to separate even the non-linearly separable data, linearly ^39, 40^.
  Initially, the algorithm will be applied using a linear kernel function, and model performance will be assessed using other kernel functions (i.e. Gaussian, sigmoid and polynomial). Depending on the model’s performance, the best kernel function will be selected. Depending on the kernel function selected, the following hyper-parameters will be regularised until the optimal prediction is made: ‘C’ value, Gamma value, degree and coefficient.

***Evaluating the model.*** Performance of each model will be evaluated using model diagnostics, and the best model will be recommended depending on the results. The trained models, as described in the previous step, will be applied to the validation dataset (30% of the data). The prediction performance of each of the models will be assessed using three methods:

1. Concordance index ^41^. The concordance index, or C-index, measures the discriminative ability of a survival model. It is defined as the fraction of pairs of patients that the patient who has a longer survival time is also predicted with lower risk score by the model. The range of concordance is between zero and one, with a larger value indicating better performance (and 0.5 indicating discrimination by chance).
2. Discriminative ability using the C-statistics for the censored function. This is the area under the receiver operating characteristics curve (ROC). The ROC curve is plotted with a sensitivity against 1–specificity, where sensitivity is on the y-axis and 1–specificity on the x-axis. The AUC ranges from 0 to 1, and a higher AUC indicates that the model is capable of distinguishing the cases (i.e. graft failure) with non-case (i.e. no graft failure).

The best performing model will be selected based on the results of the above-mentioned indicators. In an event of a discrepancy between the performance indicators, the results of concordance index will be considered as the main evaluator. We will also use other model checks such as residuals plots and testing for influential values, which may help to guide decision making about the “best” model.

Furthermore, the outputs of different machine leaning predictive models will be compared with Kidney Donor Risk Index (KDPI), a commonly used index which quantify graft failure risk before transplantation.

Data that will be used to develop the predictive models will be made available under restricted access with the permission from ANZDATA.

### Ethics

Activities of the ANZDATA registry have been granted full ethics approval by the Royal Adelaide Hospital Human Research Ethics Committee (reference number: HREC/17/RAH/408 R20170927, approval date: 28/11/2017). Even though the data is at the individual-level in the registry, only de-identified records are requested for the analysis. All electronic data will be saved with password protection on Queensland University of Technology’s secure server in encrypted folders only accessible to the nominated research staff.

## Discussion

This protocol describes the development of two separate machine learning-based predictive models to predict graft failure following live and deceased donor kidney transplant, using a large national dataset from Australia. The live donor risk prediction model will be the first machine learning based predictive model developed using a large national dataset, and the deceased donor risk prediction model will be the only machine learning based predictive model that used more than 7,000 patient records outside the United States. Furthermore, these two models will be the only two predictive models which used post kidney transplant graft survival data to model using machine learning techniques. Thus, the two predictive models are expected to provide valuable insight into the complex interactions between graft failure and donor and recipient characteristics.

The dataset necessary for the study was provided by ANZDATA. ANZDATA collects and reports the incidence, prevalence and outcome of dialysis treatment and kidney transplantation for patients with end-stage kidney disease across Australia and New Zealand. This registry started in 1977 and since then all the kidney transplant activities in Australia and New Zealand have been captured in the registry, including the transplants in the private sector. The inclusion of all kidney transplants in Australia and New Zealand, and the availability and longevity of follow-up information have been the strength of this registry ^42^. Thus, this registry has been the source of information for numerous high-impact publications ^43– 45^.

The current study proposes to use four methods, namely: Cox proportional regression, survival support vector machine, random survival forest and survival tree. The best machine learning technique available to develop a predictive model is currently being discussed widely ^46^. Most are of the view that no single technique fits all datasets, and it depends on the complexity of the data ^47^. Thus, it is imperative that different machine learning methods are used to develop predictive models on a single dataset, so that the best could be chosen using validation parameters.

This project will have some limitations. According to medical literature, there are an abundance of risk factors for graft failure following a kidney transplant. However, the proposed predictive models will be only based on the variables which have already collected by ANZDATA, thus a complete risk factor profile may not be captured. The graft failure is linked to genetic ^48^ and socio-economic factors ^49^ of the transplant population. Thus, generalisability of the proposed models to other settings outside Australia, needs to be assessed further after they have been developed.

## Data Availability

There were no underlying data associated with this article. Supplementary material : Independent variables that will be used in the models (donor and recipient characteristics) ^22^ https://doi.org/10.6084/m9.figshare.11923446.v1
