# Supplementary material for: Using machine learning techniques to develop risk prediction models to predict graft failure following kidney transplantation: protocol for a retrospective cohort study
Source: F1000Res. 2020 Mar 9;8:1810. Originally published 2019 Oct 29. [Version 2] doi: 10.12688/f1000research.20661.2 (PMC7199287; doi:10.12688/f1000research.20661.2)
Supplement: Peer review history

# Peer review: Reviewer response for version 2

**DOI:** 10.5256/f1000research.25046.r62016

**Published:** 2020-05-04

**Authors:** Slade Matthews

**Affiliations:** 1 Pharmacoinformatics Laboratory, Discipline of Pharmacology, School of Medical Sciences, Faculty of Medicine and Health, The University of Sydney, Sydney, NSW, Australia

The authors present a clear account of their approach to creating a predictive model for failure of renal transplants. The statistical and machine learning descriptions are very clear and nicely laid out. I have a few comments for the authors below:

1. In the introduction you mention that previous studies lumped together data considering live and post-mortem renal donor transplants. And that you anticipate there to be such a large difference between these two conditions that you will build two separate models for these two situations. The point is made by reviewer Chenxi Huang that you could use all the data and have an indicator variable which shows whether living or deceased donors were the organ source. Is it that the variable will be so predictive that you fear it will swamp otherwise important sources of variability in the model? Either way it is actually a testable hypothesis since you could build three models, the last one being a combined model and then answer the questions as to the deleterious contribution of this data.
2. The list of independent variables of which there are 83 includes many that would make prediction easy such as “graft failure date” and some that could be not directly related to the outcome but might give a little too much away like “age at death”. Clearly a careful consideration of what these variables mean must be had when selecting the variables allowed to comprise the model. The authors mention that “only variables available before transplantation will be used in developing prediction models” which is great and they then go on to describe feature selection methods ML & EO, PCA and EN. Then seven possible combinations are outlined in Table 1. Once these sets of descriptor variables are included in the 4 ML models will further variable selection take place such as selecting variables with significant beta coefficients in Cox Proportional Hazards Regression?
3. The authors say that the model performance evaluation will use 2 methods, the concordance index and the C-statistic. The concordance index looks at pairs of patients at a time. It compares their risk of death given by the model with the survival time recorded in the clinical data. Is this correct? So the model output is a risk of death value? I found this section a tiny bit unclear and it could benefit from an explicit description of the anticipated numerical output from the model. The 2 ^nd^ method uses the C-statistic or concordance with the ROC curve. Here the risk of graft failure predicted by the models will be subjected to a series of thresholds and sensitivity is calculated as the probability that the predicted risk value will be above the threshold for individuals for whom the graft failed. If I am correct here then I think that a sentence like this should be included because as it stands the description is somewhat terse like a man page in unix…
4. Validation of predictive machine learning models in medicine has long been a problem with the lack of clinical data availability in this setting. Have you have considered contacting the researchers in USA you mention to see if they would be willing to run their clinical data through your completed model as an external validation set. This would really add a lot of impact to the final reporting of the model performance.
5. Finally, on the whole I think this is a terrific project and I anticipate that it will achieve some really useful results. Your approach is very well thought out and with the inclusion of a couple of additional explanations more people will be able to benefit from reading this work and be inspired to adopt similar approaches in their own research.

Is the study design appropriate for the research question?

Yes

Is the rationale for, and objectives of, the study clearly described?

Yes

Are sufficient details of the methods provided to allow replication by others?

Yes

Are the datasets clearly presented in a useable and accessible format?

Not applicable

Reviewer Expertise:

I am a computational toxicologist and I have experience in machine learning applied to biomedical data and survival analysis.

I confirm that I have read this submission and believe that I have an appropriate level of expertise to confirm that it is of an acceptable scientific standard.

# Peer review: Reviewer response for version 2

**DOI:** 10.5256/f1000research.25046.r62013

**Published:** 2020-04-21

**Authors:** Chenxi Huang

**Affiliations:** 1 Center for Outcomes Research and Evaluation, Yale-New Haven Hospital, New Haven, CT, USA

The authors aimed to develop risk prediction models for graft failure following kidney transplantation using machine learning techniques. They have provided a lot of details on how the data will be prepared, what modeling techniques will be implemented and how the derived models will be compared. I have the following suggestions:

1. The major question I have is that the authors proposed to develop two separate models, one for live donor and one for deceased. Why couldn't they consider adding an indicator variable for the source of donor and build a more general model for use? Any interactions that the authors argued any single model would miss can be captured in this way. Such a model can benefit from a larger sample size and preventing overfitting. And the authors can test their hypothesis that such interaction exists.
2. The authors proposed to use a single random split with a backup plan for cross-validation. The authors should first specify what kind of cross-validation they are considering: 5-fold, 10-fold? Second, is it possible to do non-random splitting, such as splitting based on region or time of transplantation? Random data splitting often lead to failure of identifying overfitting and unrealistically optimal results.
3. The authors proposed to implement several feature selection methods. It is not clear whether they will be implemented with all modeling techniques and how many final models will be derived and compared. Also the last two feature selection methods are based on linear modeling, which does not take into account of potential interactions and linearity that the tree-based methods may be able to capture. Thus variables that may be important for subgroups may not be identified with these methods. I would suggest the authors to consider permutation methods paired with the specific machine learning modeling technique. 
4. For comparing models, the summary statistics the authors proposed to use can sometimes be uninformative. To get a more complete picture of the model performance in discrimination, suggest the authors to use decision-curve analysis or simply visualizing AUC curve at different decision points.
5. As an optional point, the authors did not consider any modeling techniques that involves neural networks. The small sample size may be why. But to be more complete in machine learning modeling techniques, could consider small networks to see if they provide any improvement.

Is the study design appropriate for the research question?

Partly

Is the rationale for, and objectives of, the study clearly described?

Yes

Are sufficient details of the methods provided to allow replication by others?

Partly

Are the datasets clearly presented in a useable and accessible format?

Not applicable

Reviewer Expertise:

biostatistics, machine learning, image analysis and processing

I confirm that I have read this submission and believe that I have an appropriate level of expertise to confirm that it is of an acceptable scientific standard, however I have significant reservations, as outlined above.

# Peer review: Reviewer response for version 2

**DOI:** 10.5256/f1000research.25046.r61037

**Published:** 2020-04-14

**Authors:** Ramkiran Gouripeddi

**Affiliations:** 1 Department of Biomedical Informatics, Center for Clinical and Translational Sciences (CCTS) Biomedical Informatics Core, University of Utah, Salt Lake City, UT, USA

I have no further comments to make.

Is the study design appropriate for the research question?

Partly

Is the rationale for, and objectives of, the study clearly described?

Partly

Are sufficient details of the methods provided to allow replication by others?

Partly

Are the datasets clearly presented in a useable and accessible format?

No

Reviewer Expertise:

Biomedical Informatics, Translational Research Informatics, Machine Learning, Data Integration, Exposome Informatics

I confirm that I have read this submission and believe that I have an appropriate level of expertise to confirm that it is of an acceptable scientific standard.

# Peer review: Reviewer response for version 1

**DOI:** 10.5256/f1000research.22723.r55930

**Published:** 2019-12-12

**Authors:** Ramkiran Gouripeddi

**Affiliations:** 1 Department of Biomedical Informatics, Center for Clinical and Translational Sciences (CCTS) Biomedical Informatics Core, University of Utah, Salt Lake City, UT, USA

Congratulations to the authors for submitting this manuscript. Here are some comments for your consideration:

1. The authors suggest using model time-to-event modeling instead of binary outcomes. Providing references to show that this is beneficial over binary outputs would be useful.
2. Modeling time-to-event would provide continuous output variables, and therefore require regression methods and not classification methods. It is not clear if the time-to-event is being model as binary variable, in which case the novelty is reduced, or the authors plan to use only regression methods. Please clarify.
3. Regarding “Three machine learning methods (survival tree, random survival forest and survival support vector machine) and one traditional regression method, Cox proportional regression, will be used to develop the two predictive models.”, is this one model live and one model for deceased donors? Changing this here and elsewhere to read as “one model live and one model for deceased donors, respectively” would make this clear.
4. “However, evidence indicate that the health systems around the world, have failed to meet the increasing demand for kidney grafts. This is evident from the growing prevalence of ESRD in the world3.” These two sentences do not seem related as suggested by the authors.
5. Listing or providing the 83 variables as a supplementary material will be helpful in reproducing methods.
6. Providing references for many of the methods mentioned (e.g. VIF) would also help.
7. “Even though having a large number of input variables may potentially produce superior results with high accuracy, in a practical sense it is important that a manageable number of input variables are used in the model.” This might not always be true. The goal is to avoid the curse of dimensionality and have appropriate number of independent variables.
8. Consider using cross-validation for model evaluation.
9. How is survival SVM different from any SVM?
10. The authors could consider evaluating the machine learning models with currently used methods in clinical settings as mentioned in the introductory remarks.

Is the study design appropriate for the research question?

Partly

Is the rationale for, and objectives of, the study clearly described?

Partly

Are sufficient details of the methods provided to allow replication by others?

Partly

Are the datasets clearly presented in a useable and accessible format?

No

Reviewer Expertise:

Biomedical Informatics, Translational Research Informatics, Machine Learning, Data Integration, Exposome Informatics,

I confirm that I have read this submission and believe that I have an appropriate level of expertise to confirm that it is of an acceptable scientific standard, however I have significant reservations, as outlined above.

# Response

**Published:** 2020-03-02

**Authors:** Sameera Senanayake

**Affiliations:** Queensland University of Technology, Australia

We are grateful for the comments made by the reviewer. They have been extremely useful for us in reviewing our protocol. We have made changes in response to the suggestions of the reviewer wherever possible and have explained our responses in detail below.

Comment 1 : The authors suggest using model time-to-event modeling instead of binary outcomes. Providing references to show that this is beneficial over binary outputs would be useful.

Response 1 : The benefit of modelling time-to-event information instead of binary information, has been included to the introduction.

*“Time-to-event (survival) information had not been modelled in any of the machine learning-based prediction models available in the medical literature, to predict graft failure after kidney transplant.  Instead, most have used the binary outcome of failure or not as the outcome variable.  However, presence of censored observations makes predictions done using this type of prediction models less accurate.  Therefore, incorporating the timing of the event to the prediction model could lead to better prediction models* *^(21)^* *.”*

Comment 2 : Modeling time-to-event would provide continuous output variables, and therefore require regression methods and not classification methods. It is not clear if the time-to-event is being model as binary variable, in which case the novelty is reduced, or the authors plan to use only regression methods. Please clarify.

Response 2 : The survival models proposed in this paper will model time-to-event data and so consider two outcomes, i.e. time and graft failure (yes/no) ^(1,2)^. This uses more information than a simple binary approach, which is statistically better than a simple binary outcome, and it uses more clinically relevant information, as patients and clinicians would like to know how long the graft will last.

*^1^* *Bou-Hamad I, Larocque D, Ben-Ameur H. A review of survival trees. Statistics Surveys. 2011;5:44-71.*

*^2^* *Banerjee M, Reyes-Gastelum D, Haymart MR. Treatment-Free Survival in Patients With Differentiated Thyroid Cancer. The Journal of Clinical Endocrinology & Metabolism. 2018 Jul;103(7):2720-7.*

Comment 3 : Regarding “Three machine learning methods (survival tree, random survival forest and survival support vector machine) and one traditional regression method, Cox proportional regression, will be used to develop the two predictive models.”, is this one model live and one model for deceased donors? Changing this here and elsewhere to read as “one model live and one model for deceased donors, respectively” would make this clear.

Response 3 : This point is well noted. Changes have been made to indicate that two separate models, one for live donors and one for deceased donors, will be developed using different data sets 

Comment 4 : “However, evidence indicates that the health systems around the world, have failed to meet the increasing demand for kidney grafts. This is evident from the growing prevalence of ESRD in the world3.” These two sentences do not seem related as suggested by the authors.

Response 4 : Please note that the final stage of chronic kidney disease is called the End Stage Renal Disease (ESRD) and patients in this stage need to either transplant a kidney or undergo dialysis to sustain life. Evidence indicates that the incidence (new patients) of ESRD is increasing around the world and if the supply of donor grafts increases accordingly, the prevalence (all patients) of ESRD is expected to reduce (as they will not be in ESRD anymore). However, the prevalence of ESRD has been increasing over recent years, indicating the demand for kidney grafts has not been met by the health systems.

Therefore, the authors believe that these 2 sentences complement each other.

Comment 5 : Listing or providing the 83 variables as a supplementary material will be helpful in reproducing methods.

Response 5 : Supplementary material indicating all possible variables has been added.

Comment 6 : Providing references for many of the methods mentioned (e.g. VIF) would also help.

Response 6 : Reference to the methods mentioned in the text has been added.

Comment 7 : Even though having a large number of input variables may potentially produce superior results with high accuracy, in a practical sense it is important that a manageable number of input variables are used in the model.” This might not always be true. The goal is to avoid the curse of dimensionality and have appropriate number of independent variables.

Response 7 : Thank you for highlighting this.  The following changes have been made to the methods section.

*“Feature (variable) selection is the process of selecting the most relevant variables that should be included in the model.  We will carefully select a potentially large set of variables to be used by the feature selection methods in discussion with clinical colleagues. We will use variable selection methods that aim to create a parsimonious set of predictor variables from the larger set using cross-validation. We will reflect on the variables selected with our clinical colleagues to verify that the model makes clinical sense.* *Since the predictive models might potentially be used in pre-transplant decision making, only variables available before transplantation will be used in developing prediction models.”*

Comment 8 : Consider using cross-validation for model evaluation.

Response 8 : Thank you for highlighting this. The following changes have been made to the methods section.

*“During model training, the dataset is randomly divided into two parts: a training dataset and validation dataset.  This prevents over-fitting and provides models that are more robust and give more realistic predictions of their prediction accuracy. Several spilt proportions have been used in models in relevant literature, such as 90:10% and 80:20%, with 70:30% being the most common* *^(30)^* *. Thus, in the present study dataset will be split into two parts, with 70% of the data to train the model and 30% to validate the developed models.* *Given our large sample size we expect that this approach would produce similar results to multiple cross-validations. However, in live donor transplant sample of around 3,758* *we will use cross-validation to estimate the variability in our model evaluation statistics, and if the variability is large (more than 10% of the mean accuracy) then we will use cross-validation for this sample.”*

Comment 9 : How is survival SVM different from any SVM?

Response 9 : Usual SVM can be used only for dichotomous outcomes and does not account to time-to-event information.  However, survival SVM accounts for time-to-event data as it considers outcomes, i.e. time and event ^(1)^.

*^1^* *Fouodo CJ, König IR, Weihs C, Ziegler A, Wright MN. Support Vector Machines for Survival Analysis with R. R Journal. 2018 Jul 1;10(1).*

Comment 10 : The authors could consider evaluating the machine learning models with currently used methods in clinical settings as mentioned in the introductory remarks.

Response 10 : Thank you for highlighting this important point. The outputs of different machine learning predictive models will be compared with Kidney Donor Risk Index (KDPI), commonly used to quantify graft failure risk before transplantation.
